# Supplementary material for: Multi‐dataset identification of innovative feature genes and molecular mechanisms in keratoconus
Source: J Cell Mol Med. 2024 Sep 19;28(18):e70079. doi: 10.1111/jcmm.70079 (PMC11412914; doi:10.1111/jcmm.70079)
Supplement: Supplementary file 2 — Material S2. [file JCMM-28-e70079-s002.zip › 7.GSEA analysis/3.DBNDD1/KEGG_SYSTEMIC_LUPUS_ERYTHEMATOSUS.html]

Details for gene set KEGG\_SYSTEMIC\_LUPUS\_ERYTHEMATOSUS[GSEA]

|  || Dataset | DBNDD1.group.cls#h\_versus\_l |
| Phenotype | group.cls#h\_versus\_l |
| Upregulated in class | l |
| GeneSet | KEGG\_SYSTEMIC\_LUPUS\_ERYTHEMATOSUS |
| Enrichment Score (ES) | -0.50067097 |
| Normalized Enrichment Score (NES) | -1.212146 |
| Nominal p-value | 0.27027026 |
| FDR q-value | 0.7006122 |
| FWER p-Value | 0.988 |
Table: GSEA Results Summary

  

Fig 1: Enrichment plot: KEGG\_SYSTEMIC\_LUPUS\_ERYTHEMATOSUS      
 Profile of the Running ES Score & Positions of GeneSet Members on the Rank Ordered List

  

| SYMBOL | TITLE | RANK IN GENE LIST | RANK METRIC SCORE | RUNNING ES | CORE ENRICHMENT || 1 | CD86 | na | 1030 | 0.379 | 0.0111 | No |
| 2 | HLA-DRB5 | na | 1509 | 0.335 | 0.0334 | No |
| 3 | FCGR1A | na | 3835 | 0.219 | -0.0100 | No |
| 4 | ELANE | na | 5104 | 0.177 | -0.0275 | No |
| 5 | C8A | na | 5449 | 0.166 | -0.0196 | No |
| 6 | HLA-DMA | na | 8201 | 0.100 | -0.0882 | No |
| 7 | C6 | na | 8447 | 0.096 | -0.0849 | No |
| 8 | FCGR2A | na | 8721 | 0.090 | -0.0831 | No |
| 9 | FCGR2B | na | 8722 | 0.090 | -0.0733 | No |
| 10 | HLA-DRB4 | na | 10303 | 0.067 | -0.1116 | No |
| 11 | C1QA | na | 10386 | 0.066 | -0.1069 | No |
| 12 | HLA-DRB1 | na | 10490 | 0.065 | -0.1029 | No |
| 13 | GRIN2A | na | 10969 | 0.059 | -0.1104 | No |
| 14 | HLA-DOA | na | 11835 | 0.049 | -0.1301 | No |
| 15 | HLA-DQB1 | na | 12864 | 0.039 | -0.1555 | No |
| 16 | ACTN4 | na | 12890 | 0.039 | -0.1520 | No |
| 17 | C9 | na | 13377 | 0.035 | -0.1622 | No |
| 18 | C1QC | na | 14542 | 0.026 | -0.1930 | No |
| 19 | SNRPD1 | na | 16511 | 0.013 | -0.2484 | No |
| 20 | ACTN2 | na | 17576 | 0.006 | -0.2784 | No |
| 21 | C8B | na | 17744 | 0.005 | -0.2827 | No |
| 22 | C7 | na | 19181 | -0.006 | -0.3235 | No |
| 23 | SSB | na | 19551 | -0.009 | -0.3332 | No |
| 24 | HLA-DOB | na | 21192 | -0.024 | -0.3779 | No |
| 25 | SNRPB | na | 21223 | -0.024 | -0.3762 | No |
| 26 | CD40 | na | 21562 | -0.028 | -0.3830 | No |
| 27 | CD28 | na | 21937 | -0.031 | -0.3904 | No |
| 28 | SNRPD3 | na | 22046 | -0.033 | -0.3900 | No |
| 29 | C8G | na | 23047 | -0.044 | -0.4141 | No |
| 30 | TRIM21 | na | 24349 | -0.061 | -0.4450 | No |
| 31 | CTSG | na | 24437 | -0.062 | -0.4408 | No |
| 32 | HLA-DRB3 | na | 25022 | -0.071 | -0.4500 | No |
| 33 | HLA-DRA | na | 26179 | -0.091 | -0.4735 | No |
| 34 | GRIN2B | na | 26883 | -0.105 | -0.4825 | Yes |
| 35 | HLA-DMB | na | 26948 | -0.107 | -0.4728 | Yes |
| 36 | CD80 | na | 27049 | -0.109 | -0.4640 | Yes |
| 37 | TNF | na | 27164 | -0.111 | -0.4552 | Yes |
| 38 | FCGR3A | na | 27389 | -0.116 | -0.4492 | Yes |
| 39 | HLA-DPA1 | na | 28024 | -0.132 | -0.4533 | Yes |
| 40 | FCGR2C | na | 29669 | -0.178 | -0.4815 | Yes |
| 41 | C5 | na | 29749 | -0.181 | -0.4643 | Yes |
| 42 | ACTN1 | na | 30298 | -0.197 | -0.4588 | Yes |
| 43 | C3 | na | 30316 | -0.198 | -0.4380 | Yes |
| 44 | C4B | na | 31150 | -0.226 | -0.4376 | Yes |
| 45 | IL10 | na | 31214 | -0.229 | -0.4147 | Yes |
| 46 | CD40LG | na | 31492 | -0.240 | -0.3969 | Yes |
| 47 | C1R | na | 32098 | -0.269 | -0.3853 | Yes |
| 48 | HLA-DPB1 | na | 32889 | -0.320 | -0.3737 | Yes |
| 49 | C1QB | na | 33014 | -0.329 | -0.3417 | Yes |
| 50 | C1S | na | 33313 | -0.354 | -0.3122 | Yes |
| 51 | C2 | na | 33504 | -0.371 | -0.2777 | Yes |
| 52 | IFNG | na | 33717 | -0.393 | -0.2414 | Yes |
| 53 | ACTN3 | na | 33884 | -0.415 | -0.2014 | Yes |
| 54 | C4A | na | 33988 | -0.428 | -0.1583 | Yes |
| 55 | HLA-DQA2 | na | 34710 | -0.832 | -0.0894 | Yes |
| 56 | HLA-DQA1 | na | 34711 | -0.834 | 0.0005 | Yes |
Table: GSEA details [plain text format]

  

Fig 2: KEGG\_SYSTEMIC\_LUPUS\_ERYTHEMATOSUS      
 Blue-Pink O' Gram in the Space of the Analyzed GeneSet

  

Fig 3: KEGG\_SYSTEMIC\_LUPUS\_ERYTHEMATOSUS: Random ES distribution      
 Gene set null distribution of ES for **KEGG\_SYSTEMIC\_LUPUS\_ERYTHEMATOSUS**
